# Mitochondrial Dysfunction: The Cellular Bridge from Emotional Stress to Disease Onset: A Narrative Review

**DOI:** 10.3390/biom16010117

**Published:** 2026-01-08

**Authors:** Sakthipriyan Venkatesan, Cristoforo Comi, Fabiola De Marchi, Teresa Esposito, Carla Gramaglia, Carlo Smirne, Mohammad Mostafa Ola Pour, Mario Pirisi, Rosanna Vaschetto, Patrizia Zeppegno, Elena Grossini

**Affiliations:** 1 Laboratory of Physiology, Department of Translational Medicine, Università del Piemonte Orientale, via Solaroli 17, 28100 Novara, Italy; sakthipriyan.venkatesan@uniupo.it (S.V.); 20046522@studenti.uniupo.it (M.M.O.P.); 2 Neurology Unit, Department of Translational Medicine, Università del Piemonte Orientale, via Solaroli 17, 28100 Novara, Italy; cristoforo.comi@med.uniupo.it (C.C.); fabiola.demarchi@uniupo.it (F.D.M.); 3 Anesthesiologist Unit, Department of Translational Medicine, Università del Piemonte Orientale, via Solaroli 17, 28100 Novara, Italy; expoterry@gmail.com (T.E.); rosanna.vaschetto@med.uniupo.it (R.V.); 4 Psychiatric Unit, Department of Translational Medicine, Università del Piemonte Orientale, via Solaroli 17, 28100 Novara, Italy; carla.gramaglia@med.uniupo.it (C.G.); patrizia.zeppegno@med.uniupo.it (P.Z.); 5 Internal Medicine Unit, Department of Translational Medicine, Università del Piemonte Orientale, via Solaroli 17, 28100 Novara, Italy; carlo.smirne@med.uniupo.it (C.S.); mario.pirisi@uniupo.it (M.P.)

**Keywords:** allostatic load, mitochondrial dysfunction, psychosocial stress, relationship trauma, reactive oxygen species, systemic nervous system

## Abstract

Severe emotional stress constitutes a significant public-health concern associated with negative health outcomes. Although the clinical effects are well acknowledged, the specific biological mechanisms that translate emotional suffering into systemic disease remain incompletely understood. Psychological stress activates the sympathetic nervous system and hypothalamic–pituitary–adrenal axis, which directly target mitochondria and alter their bioenergetic and redox capacity. For this reason, this narrative review proposes that mitochondria serve as the primary subcellular link in the mind–body connection, as they play a pivotal role in converting neuroendocrine signals into cellular dysfunction. In particular, we focus on the concept of mitochondrial allostatic load (MALT), a framework explaining how the progressive decline in mitochondrial functions, from their initial adaptive roles in energy production, reactive oxygen species signaling, and calcium regulation, to being sources of inflammation and systemic damage, occurs when stress exceeds regulatory limits. We also, discuss how this transition turns mitochondria from adaptive responders into drivers of multi-organ disease. In subsequent sections, we examine diagnostic potentials related to MALT, including the use of biomarkers, such as growth differentiation factor 15, cell-free mitochondrial desoxyribonucleic acid, and functional respirometry. Furthermore, we evaluate mitochondria-targeted therapeutic strategies, encompassing pharmacological compounds, such as mitoquinone mesylate, Skulachev ions, and elamipretide, alongside lifestyle and psychological interventions. Here, we aim to translate MALT biology into clinical applications, positioning mitochondrial health as a target for preventing and treating stress-related disorders. We propose that MALT may serve as a quantifiable bridge between emotional stress and somatic disease, enabling future precision medicine strategies integrating mitochondrial care.

## 1. Introduction

Severe emotional stress resulting from loss, bereavement, or trauma is consistently associated with increased risk of cardiovascular disease, metabolic disorders, psychiatric conditions, and higher rates of morbidity and mortality [1,2]. These outcomes create a substantial burden on individuals and healthcare systems worldwide. Although the epidemiological link between psychosocial stress and adverse health outcomes is well documented, the biological pathways through which psychological experiences produce lasting physical effects remain incompletely understood [3]. Psychological stress activates two interconnected neuroendocrine systems, namely the sympathetic nervous system (SNS) and the hypothalamic–pituitary–adrenal (HPA) axis [4]. Activation of these pathways leads to the release of catecholamines and cortisol, hormones that can mobilize metabolic resources and enhance survival [5,6]. Nevertheless, frequent or chronic activation of these signals has the potential to disrupt cardiovascular, metabolic, and immune regulation [7].

Despite decades of research, a critical question remains regarding how emotional experiences yield persistent changes at the level of tissues and cells. While the roles of stress hormones and immune mediators are well established, the subcellular mechanisms that respond to and “record” psychological stress are only beginning to be mapped [8]. Mitochondria, which are essential organelles responsible for energy production, redox homeostasis and calcium signaling are emerging as important players in this process [9]. Furthermore, they express receptors for stress hormones, such as glucocorticoids and catecholamines, rapidly adjust to fluctuations in calcium and metabolic state, and can adapt to acute challenges [10,11]. It is also apparent that under conditions of repeated or sustained stress, these adaptive responses may become overwhelmed or dysregulated [12].

Building on McEwen’s concept of allostatic load, which describes the cumulative biological burden of stress [13], we propose that a related process unfolds within mitochondria. This hypothesis, commonly referred to as mitochondrial allostatic load (MALT), suggests that emotional stress gradually alters both mitochondrial function and structure [14]. In the context of acute emotional stress, mitochondria may increase energy output and activate antioxidant defenses. Over time, however, when emotional stress is sustained or recurrent, mitochondria activation may precipitate their dysfunction, which is characterized by reduced energy production, increased reactive oxygen species (ROS) formation, perturbed calcium handling, mitochondrial fragmentation, and the release of mitochondrial components, such as mitochondrial deoxyribonucleic acid (DNA) (mtDNA), into the cell or bloodstream [15,16]. These events can promote inflammation and alter gene regulation, thereby creating a form of cellular memory that influences health long after resolution of the initial stressor [17]. If this model is valid, it could help to clarify how emotional stress may drive long-term health effects and why these effects may sometimes persist for years. This framework may also provide new tools to identify individuals at risk and enable more effective, targeted interventions.

Critically, understanding the cellular and molecular mechanisms of MALT necessitates the development of measurable biomarkers that can track mitochondrial dysfunction in response to emotional stress. In this review, we examine evidence for stress-related changes in mitochondrial biomarkers, including growth differentiation factor 15 (GDF-15), cell-free mitochondrial DNA, and high-resolution respirometry [18,19]. In addition, we summarize current and emerging therapeutic strategies that specifically target mitochondrial function, encompassing pharmacological agents (for example, mitoquinone (MitoQ), Skulachev ions (SkQs), and elamipretide (SS-31)), as well as lifestyle and psychological interventions [20,21]. Overall, our review brings together epidemiological findings, mechanistic studies, and translational opportunities to explore how emotional stress may be embedded at the subcellular level. Our aim is to clarify the potential role of mitochondria in the mind–body interface, assess the state of the knowledge, and outline directions for future research and therapy, while acknowledging the complexity of these pathways and the need for further clinical validation.

## 2. Materials and Methods

This narrative review was conducted by searching the PubMed, Scopus, and Web of Science databases for articles published up to September 2025. The search strategy utilized combinations of the following keywords: “Psychosocial Stress,” “Mitochondria,” “Allostatic Load,” “Glucocorticoids,” “ROS,” “HPA axis,” “Mitochondrial Dynamics,” and “Inflammation.” Inclusion criteria encompassed peer-reviewed studies in English reporting human or animal data on stress-mitochondria interactions, biomarkers, and therapeutics. Exclusion criteria removed non-peer-reviewed sources and purely genetic mitochondrial disorders. We also manually reviewed the reference lists of identified articles to ensure a comprehensive synthesis of the available literature.

## 3. Foundational Concepts

Understanding MALT requires engagement with three established domains: severe psychosocial stress and its epidemiological consequences, the allostatic load framework that operationalizes systemic wear-and-tear, and mitochondrial psychobiology, which places mitochondria as integrators of stress signals at the molecular level. We synthesize these domains below.

### 3.1. Severe Psychosocial Stress and Neuroendocrine Temporal Dynamics

Severe psychosocial stress arising from relationship loss, bereavement, trauma, discrimination, or acute socioeconomic adversity represents a distinct category of psychological challenge, consistently ranked among life’s most consequential adverse events [1,2]. The phenomenology and neuroendocrine response of severe stress unfolds temporally in distinct phases. Acute phases, spanning days to weeks, are characterized by intense emotional pain, hypervigilance, and robust activation of both the HPA axis and the SNS [22]. During these acute phases, catecholamine release from the adrenal medulla and corticotropin-releasing hormone (CRH) release from the hypothalamus converge to drive cortisol secretion from the adrenal cortex [4,23]. Conversely, chronic phases that extend over months to years, when stressors persist or recur, manifest markedly differently. This stage is characterized by persistent low-grade inflammation mediated by elevated interleukin-6 (IL-6), tumor necrosis factor alpha (TNF-α), and other pro-inflammatory cytokines, alongside neuroendocrine dysregulation and psychiatric sequelae, such as depression and post-traumatic stress disorder (PTSD) [24,25]. Epidemiologically, severe emotional stress is associated with a substantially increased risk of cardiovascular disease, metabolic dysfunction, psychiatric illness, and all-cause mortality. These associations reflect biologically mediated pathways rooted in dysregulation of neuroendocrine signaling and chronic neuroinflammation, underscoring the critical need to understand mechanisms by which psychological adversity becomes physiologically embedded.

### 3.2. Allostatic Load: From Adaptation to Maladaptation

McEwen’s concept of allostasis describes the dynamic process through which organisms maintain physiological stability in response to environmental demands via coordinated neuroendocrine, autonomic, and immune signaling [13]. Allostatic load refers to the cumulative biological burden imposed by repeated, chronic, or severe stress, reflecting the physiological cost incurred when adaptive responses are sustained beyond their functional utility [15]. Under acute challenges, allostasis functions protectively through HPA axis-mediated glucocorticoid signaling and SNS-driven catecholamine release, which mobilize energy substrates and enhance survival. However, sustained or recurrent activation produces measurable dysregulation, including abnormal cortisol dynamics (such as elevated baseline or flattened diurnal rhythms), elevated pro-inflammatory markers (IL-6, TNF-α, C-reactive protein), altered glucose metabolism, and cardiovascular dysfunction including hypertension and atherosclerosis [16]. Critically, allostatic load is quantifiable through objective biomarkers. The key insight is that allostatic load represents a transition from adaptive to maladaptive states; thus, mechanisms that initially protect the organism become, with persistent activation, a source of pathology. A limitation of current allostatic load frameworks is their focus on whole-organism and tissue-level phenomena, leaving unanswered how this adaptive-to-maladaptive transition occurs at the subcellular level. This gap motivates operationalizing allostatic load specifically at the mitochondrial level.

### 3.3. Mitochondrial Stress Sensing and the Transition from Adaptive to Pathological Responses

Mitochondria function as far more than passive adenosine triphosphate (ATP)-producing organelles. At the molecular level, they integrate multiple stress signals through direct receptors and metabolic sensing mechanisms. Glucocorticoids bind to mitochondrial glucocorticoid receptors (mGRs), altering the expression of electron transport chain (ETC) complexes and respiratory capacity [10,26]. Similarly, catecholamines bind to β-adrenergic receptors on the mitochondrial surface, activating adenylyl cyclase and increasing cyclic adenosine monophosphate (cAMP) levels, which phosphorylates and activates mitochondrial kinases including calcium-calmodulin-dependent protein kinase II (CaMKII) [11,27].

Under acute stress, these signals trigger adaptive mitochondrial responses. Calcium influx into mitochondria via the mitochondrial calcium uniporter (MCU) drives Krebs’s cycle dehydrogenases and increases ETC flux, thereby boosting ATP synthesis in response to energy demand [12]. The controlled production of ROS, particularly hydrogen peroxide at complexes I and III, functions as a second messenger that oxidizes cysteine residues on Kelch-like ECH-associated protein 1 (KEAP1); this inactivation releases Nrf2, allowing it to induce the transcription of protective antioxidant enzymes including superoxide dismutase (SOD) and catalase [28]. Furthermore, mitochondrial dynamics respond to stress through the phosphorylation of dynamin-related protein 1 (Drp1) and regulation of mitofusins (Mfn1/Mfn2) and optic atrophy 1 (Opa1), maintaining a fusion-biased state that optimizes bioenergetic efficiency and functional complementation [29]. These acute adaptive responses activate quality control mechanisms, including mitophagy, which selectively clears damaged organelles. This adaptive phase is self-limiting; once the stressor resolves, mitochondrial function normalizes.

However, when emotional stress is sustained or recurrent, these same adaptive mechanisms become overwhelmed and transition to maladaptive states. Persistent glucocorticoid and catecholamine signaling exhausts antioxidant capacity, leading to excessive ROS accumulation that damages lipids, proteins, and mtDNA [30]. Sustained calcium influx triggers pathological calcium overload, opening the mitochondrial permeability transition pore (mPTP) and collapsing the membrane potential. Chronic Drp1 phosphorylation and suppression of fusion machinery drive excessive mitochondrial fragmentation, isolating damaged organelles from functional complementation and overwhelming mitophagy capacity [31]. Impaired ETC function reduces ATP synthesis despite continued metabolic demand, creating a bioenergetic crisis. Finally, breached mitochondrial integrity releases mtDNA and cardiolipin into the cytosol. These molecules serve as damage-associated molecular patterns (mtDAMPs) that activate innate immune pathways, including the cyclic guanosine monophosphate (GMP)-AMP synthase-stimulator of interferon genes (cGAS-STING) and NOD-like receptor family pyrin domain containing 3 (NLRP3) inflammasome pathways, perpetuating systemic inflammation [17,32]. These three domains converge at the molecular level to establish a critical principle: mitochondria have a finite capacity to buffer and adapt to stress signals. When this threshold is exceeded, they shift from adaptive responders to drivers of pathology. This principle motivates a new framework that operationalizes the subcellular manifestation of allostatic load and explains how emotional stress becomes biologically embedded through progressive mitochondrial dysfunction.

## 4. MALT: A Subcellular Framework

The concept of allostatic load describes the cumulative physiological toll of chronic stress adaptation across multiple organ systems [13]. However, the subcellular substrate underlying this wear-and-tear has remained poorly defined. MALT proposes that mitochondria serve as a key integrator of emotional stress exposure, wherein cumulative adversity progressively depletes bioenergetic, redox, and quality control reserves. This depletion ultimately shifts the organelle from a state of adaptive responsiveness to one of pathological signaling [14].

**Figure 1:**
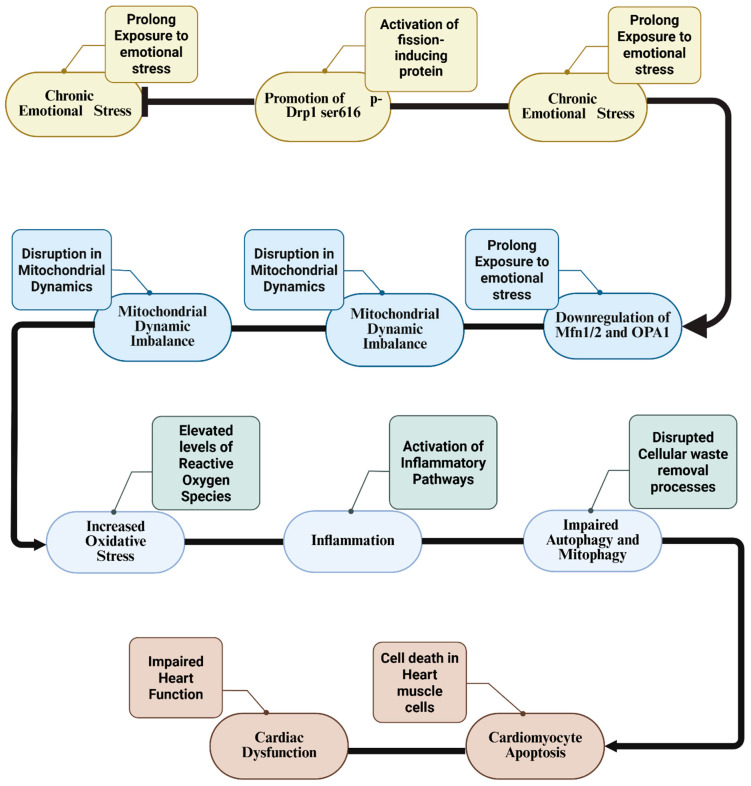
Molecular mechanisms linking chronic emotional stress to mitochondrial dysfunction and cardiomyocyte pathology. This flowchart delineates the signaling pathway by which sustained emotional stress triggers mitochondrial fragmentation. Chronic stress leads to the phosphorylation of Drp1 at Ser616 (promoting fission) and the downregulation of Mfn1/2 and OPA1 (impairing fusion). This dynamic imbalance results in increased ROS production and inflammation, which subsequently impairs autophagy and mitophagy. The accumulation of damaged organelles ultimately triggers cardiomyocyte apoptosis and cardiac dysfunction. Drp1: Dynamin-related protein 1; Mfn1/2: Mitofusin 1 and 2; OPA1: Optic atrophy 1; p-Drp1: Phosphorylated Dynamin-related protein 1; ROS: Reactive Oxygen Species. Created in BioRender. https://BioRender.com/ygum0hj (accessed on 10 December 2025).

MALT conceptualizes mitochondrial stress responses along a dynamic continuum. While the initial neuroendocrine activation triggers adaptive responses, such as calcium-driven ATP synthesis, Nrf2-mediated antioxidant defense, and fusion-biased dynamics, these mechanisms are typically reversible and self-limiting in the acute phase [12,28,29]. MALT operationalizes the cumulative cost of maintaining this high-energy state. It postulates that when stress is repeated or prolonged, critical mitochondrial “buffers” (e.g., antioxidant enzymes, spare respiratory capacity, mitophagy potential) eventually become exhausted. Once these limits are crossed, the system bifurcates from adaptation to decompensation: controlled ROS signaling shifts to oxidative damage, transient calcium fluxes become pathological overload, triggering mPTP opening, and fusion shifts to irreversible fragmentation [33]. This framework moves beyond listing molecular events to identifying the specific tipping point where mitochondrial resilience fails, potentially turning the organelle into a source of systemic inflammation via DAMP release [17]. The molecular cascade linking chronic emotional stress to mitochondrial dynamic imbalance, subsequent cellular dysfunction and cardiomyopathy is illustrated in Figure 1.

MALT extends existing frameworks by operationalizing allostatic load at the organelle level, integrating fragmented stress-response mechanisms into a unified cascade, and reframing therapeutic strategy around mitochondrial resilience. It proposes that mitochondrial reserve capacity, as assessed through high-resolution respirometry, circulating biomarkers such as GDF-15 and cell-free mitochondrial DNA (cf-mtDNA), and metabolic flux analysis, may determine an individual’s systemic resilience to psychosocial stress [18,19]. This shifts focus from cataloging whole-organism dysregulation to identifying a specific subcellular bottleneck. The framework further unifies previously siloed observations by showing that stress hormone–induced fission, cytosolic mtDNA release, NLRP3 inflammasome activation by oxidized cardiolipin, and epigenetic reprogramming via altered availability of mitochondrial metabolites such as acetyl-coenzyme A (CoA) and α-ketoglutarate are positioned as sequential, interdependent stages of a single pathological trajectory [32,34]. This integration helps explain the limited efficacy of interventions targeting isolated downstream effectors, such as interleukin 1 (IL-1)β blockade or generic antioxidant therapy, since compensatory activation of parallel pathways may persist when core mitochondrial dysfunction remains unaddressed. Instead, MALT suggests that restoring mitochondrial functional capacity through mitochondrial-targeted antioxidants, respiratory support, metabolic flexibility enhancement, or stimulation of biogenesis may be necessary to interrupt the central driver of systemic dysregulation.

This model is consistent with, yet distinct from, mitochondrial psychobiology, which established that stress hormones directly modulate mitochondrial function via glucocorticoid and β-adrenergic receptors [10]. MALT builds on this foundation by specifying the conditions under which such modulation becomes maladaptive, specifically when stress intensity, duration, or recurrence exceeds the organelle’s compensatory capacity. It also differs fundamentally from primary mitochondrial diseases (PMDs), which arise from genetic mutations in oxidative phosphorylation components and manifest as intrinsic, progressive bioenergetic failure from early life. Notably, individuals with PMDs do not consistently exhibit elevated rates of trauma-related psychopathology, depression, anxiety, or PTSD, despite severe impairments in ATP production and oxidative metabolism [35]. This dissociation implies that the neuropsychiatric consequences of MALT reflect not generic bioenergetic insufficiency but the specific inflammatory and epigenetic sequelae of stress hormone–driven mitochondrial signaling, including glucocorticoid receptor activation, mtDNA–cGAS-STING engagement, and cardiolipin–NLRP3 inflammasome assembly.

Support for MALT is robust in preclinical models. Chronic social defeat, early-life neglect, and repeated restraint stress consistently induce mitochondrial fragmentation, reduced oxidative phosphorylation capacity, mtDNA release, and systemic inflammation across rodent, primate, and avian species [36,37]. Furthermore, specific mutant mouse models demonstrate that defects in mitochondrial respiration alter the organism’s physiological response to psychological stress, confirming the bidirectional link between mitochondrial function and stress adaptation [38]. In humans, circulating biomarkers such as GDF-15, cf-mtDNA, and 8-oxo-2′-deoxyguanosine (8-oxo-dG) correlate with trauma exposure, predict all-cause morbidity and mortality in population cohorts, and are elevated in individuals with PTSD or depression. However, these associations are largely cross-sectional and subject to significant confounding from physical inactivity, poor sleep, diet, psychotropic medications, and comorbid illness. Critically, no prospective longitudinal study has yet tracked mitochondrial function before and after trauma exposure while simultaneously assessing psychological stress, systemic inflammation, and incident disease, leaving causal inference unsubstantiated.

MALT remains a testable hypothesis rather than established doctrine. Its value lies in generating falsifiable predictions: first, that baseline mitochondrial reserve capacity predicts individual vulnerability to trauma-related illness; second, that mitochondrial-targeted interventions, including pharmacological agents (e.g., MitoQ, SS-31), metabolic strategies (e.g., structured exercise, ketogenic diet), and trauma-focused psychotherapy, reduce systemic inflammation, normalize mitochondrial biomarkers, and lower disease incidence [20,21]; third, that organ-specific mitochondrial dysfunction underlies the multi-system manifestations of trauma, including stress cardiomyopathy, accelerated type 2 diabetes, depression, and autoimmune dysregulation. Definitive validation will require prospective cohorts enrolling individuals before or immediately after trauma with serial assessment of mitochondrial function (e.g., high-resolution respirometry in peripheral cells), standardized biomarker panels, validated psychological measures, and comprehensive lifestyle phenotyping.

## 5. Pathophysiological Mechanisms and Clinical Manifestations

Having established MALT as a theoretical framework, we will now examine the mechanistic evidence, drawn mainly from animal models and human correlation studies, that links emotional stress to mitochondrial dysfunction and systemic diseases. We will emphasize both established findings and speculative mechanisms, acknowledge evidence gaps, and consider alternative explanations and reverse causality.

### 5.1. Neuro-Hormonal Responses and Initial Mitochondrial Engagement

Severe emotional stress may engage a distributed neural pain network, including regions like the dorsal anterior cingulate cortex and anterior insula, which are implicated in integrating sensory, emotional, and interoceptive signals [22,39]. These cortical regions receive projections from the amygdala, hippocampus, and prefrontal cortex, enabling integration of threat appraisal, emotional significance, and memory [40]. The resulting activation pattern, characterized by heightened salience attribution and threat anticipation, appears to drive rapid output to the periaqueductal gray, a brainstem structure involved in fight-or-flight responses and HPA axis activation through hypothalamic projections [41].

Within minutes of perceiving overwhelming loss or threat, this neurobiological cascade engages two major neuroendocrine axes: one involving the HPA axis and the other involving catecholamine release. CRH neurons in the paraventricular hypothalamus increase firing, driving pituitary release of adrenocorticotropic hormone, which stimulates cortisol secretion from the adrenal cortex [4]. Simultaneously, the PAG activates the locus coeruleus and sympathetic preganglionic neurons innervating the adrenal medulla, triggering the release of norepinephrine and epinephrine [22]. This dual activation creates sustained elevation of both cortisol (minutes to hours duration) and catecholamines (seconds to minutes), establishing a neuroendocrine milieu that directly engages mitochondrial signaling. Hence, mitochondria possess functional receptors for these stress hormones, enabling direct transduction of neuroendocrine signals at the subcellular level [42]. Increasing evidence indicates that catecholamines can bind to β-adrenergic receptors on the mitochondrial outer membrane, activating adenylyl cyclase and increasing intracellular cAMP [11,27]. Elevated cAMP, in turn, may activate protein kinase A, which phosphorylates mitochondrial targets including CaMKII, enhancing mitochondrial calcium responsiveness [31]. Also, cortisol can bind to mGRs, which mediate rapid, non-genomic effects distinct from those caused by nuclear glucocorticoid receptor signaling [10,26]. Notably, activation of mGRs appears to enhance expression of ETC complexes I through IV and ATP synthase, increasing mitochondrial bioenergetic capacity and oxygen consumption rate [43].

Emotional stress, as an acute event, simultaneously drives calcium influx into the cytoplasm through voltage-gated calcium channels and release from intracellular stores. Mitochondria sequester this calcium via the MCU, a selective channel on the inner mitochondrial membrane [44]. Calcium entry into the mitochondrial matrix directly activates three rate-limiting Krebs’s cycle enzymes: isocitrate dehydrogenase, α-ketoglutarate dehydrogenase, and malate dehydrogenase [45]. Enhanced Krebs’s cycle flux increases production of nicotinamide adenine dinucleotide and flavin adenine dinucleotide, which are oxidized by the ETC, driving proton pumping and increasing the proton-motive force. The net result is an acute surge in ATP synthesis, calibrated to support elevated metabolic demands of emotional stress responses (e.g., increased muscle contraction, enhanced neurotransmitter synthesis, accelerated immune cell trafficking) [46]. Concomitantly, controlled ROS production occurs at ETC complexes I and III, primarily in the form of superoxide anion and hydrogen peroxide [28]. At physiological transient concentrations, hydrogen peroxide functions as a second messenger, diffusing across membranes to oxidize critical cysteine residues on KEAP1, a negative regulator of the transcription factor, Nrf2 [47]. KEAP1 inactivation releases Nrf2, which translocates to the nucleus, binds ARE, and activates transcription of cytoprotective genes including SOD1 and SOD2, catalase, glutathione S-transferases, and glutathione peroxidase [48]. This ROS-Nrf2-ARE axis functions as a feedback-regulated antioxidant response, wherein mild ROS production triggers expression of the very antioxidants that neutralize excess ROS.

Mitochondrial dynamics adjust to emotional stress in the acute phase through upregulation of fusion proteins (Opa1, Mfn1, Mfn2) and inactivation of the fission protein Drp1 [29]. This fusion-biased network state allows rapid redistribution of metabolites and ions between interconnected mitochondria, maximizing ATP production and enabling functional complementation, where a healthy mitochondrion can provide ATP or calcium buffering to a transiently stressed neighbor. At the same time, quality control mechanisms are activated, and damaged mitochondria are tagged and selectively removed through mitophagy, ensuring only functional organelles remain in the mitochondrial pool [49]. Critically, the acute adaptive phase is self-limiting. Once the stressor is removed, catecholamine and cortisol levels drop, calcium influx ceases, ROS production returns to basal levels, and mitochondrial dynamics rebalance toward homeostasis. Recovery occurs on the timescale of hours to days as antioxidant defenses neutralize residual ROS, damaged proteins are cleared, and the system restabilizes. In principle, this reversible adaptation should protect the organism; instead, persistent or recurrent stress exposure may alter this outcome.

### 5.2. Transition to Chronic Mitochondrial Dysfunction

The mechanistic transition from adaptive to pathological mitochondrial states occurs when emotional stress is sustained, recurrent, or persistently intense. Animal models of chronic emotional stress, early-life adversity, and repeated trauma consistently demonstrate that prolonged stress exposure leads to progressive mitochondrial dysfunction, including elevated ROS production, fission-biased remodeling, reduced ATP synthesis capacity, and mtDNA release [50,51]. However, whether this mechanistic transition occurs in humans exposed to severe emotional trauma, and the precise timeline and threshold for this transition, remain incompletely characterized. The theoretical transition point involves exhaustion of three critical mitochondrial reserves. First, antioxidant defense systems are finite. Continuous catecholamine and cortisol elevation drives constitutive ROS production, consuming SOD, catalase, glutathione, and thioredoxin pools faster than they can be replenished [52]. Cross-sectional studies show associations between trauma exposure and reduced circulating antioxidant capacity, consistent with this mechanism, though causality is not established [53]. Second, the ETC has a maximum ATP production rate; under chronically elevated energy demand, the ETC operates near maximum capacity, leaving minimal “spare respiratory capacity,” which is the ability to increase respiration in response to additional acute stressors. Depletion of spare capacity eliminates bioenergetic resilience [54]. Third, mitophagy and autophagy have finite capacity; chronic mitochondrial damage can saturate these quality control pathways, leading to accumulation of dysfunctional organelles [55].

When these reserves are depleted, a bifurcation is theorized to occur, where the mitochondrion shifts from compensation to decompensation. Superoxide anion is no longer efficiently neutralized by depleted SOD, instead accumulating and reacting with nitric oxide to form peroxynitrites, a highly oxidizing species that damages proteins, lipids, and mtDNA [30,56]. Accumulated oxidative damage impairs electron transport efficiency, further reducing ATP synthesis despite continued (albeit inefficient) substrate oxidation. This creates a vicious cycle where impaired ATP synthesis reduces energy available for antioxidant enzyme activity, permitting further ROS accumulation. Sustained calcium influx, initially adaptive, may become pathological when mitochondrial calcium buffering capacity is exceeded [34]. Excessive calcium accumulation triggers opening of the mPTP, a high-conductance channel formed by voltage-dependent anion channel (VDAC), adenine nucleotide translocator (ANT), and cyclophilin D [33]. mPTP opening dissipates the membrane potential, collapses ATP synthesis, and releases cytochrome c and other pro-apoptotic factors into the cytosol, initiating intrinsic apoptosis if damage is irreversible [57].

Chronic elevation of Drp1 phosphorylation and suppression of Opa1 shift the fusion-fission balance dramatically toward excessive fragmentation [58]. Fragmented mitochondria cannot receive complementary factors from the network and are more susceptible to mitophagy recognition, yet when mitophagy is saturated, they accumulate. These residual dysfunctional mitochondria continue to leak electrons and produce excessive ROS, perpetuating the oxidative damage cascade. An important consideration is whether reverse causality explains the mitochondrial changes seen in stressed individuals. Could systemic inflammation, sedentary behavior, poor sleep quality, malnutrition, or other common consequences of severe trauma secondarily impair mitochondrial function? While chronic mitochondrial dysfunction may contribute to symptomatology observed after emotional stress, it remains unclear whether trauma-induced mitochondrial decline is the primary driver or an epiphenomenon of broader lifestyle and physiological dysregulation.

### 5.3. Mitochondria-Derived Inflammatory Signaling

Breaching of mitochondrial membrane integrity during pathological MALT releases mtDNA and cardiolipin into the cytosol and extracellular space. MtDNA is evolutionarily derived from bacterial DNA, retaining unmethylated Cytosine-phosphate-Guanine (CpG) dinucleotides and bacterial-like structural features [17]. Pattern recognition receptors, particularly Toll-like receptor 9 and the inflammasome component NLRP3, recognize mtDNA as a pathogen-associated molecular pattern, triggering innate immune activation despite its endogenous origin. The cGAS-STING pathway represents one key mechanism. Cytosolic mtDNA is sensed by cGAS, which synthesizes cyclic GMP-AMP (cGAMP) [59]. cGAMP activates STING, recruiting and activating tank-binding kinase 1, which phosphorylates interferon regulatory factor 3 (IRF3). Phosphorylated IRF3 translocates to the nucleus and drives transcription of type I interferon genes (IFN-β, IFN-α), establishing a pro-inflammatory, antiviral-like state in the absence of viral infection [60]. This cGAS-STING-IRF3 axis may contribute to the sustained interferon-like immunological signatures observed in some PTSD cohorts [61].

The NLRP3 inflammasome represents a second key pathway. Oxidized cardiolipin translocates from the inner to the outer mitochondrial membrane during stress, where it directly activates NLRP3 [62]. The NLRP3 inflammasome assembly recruits pro-caspase-1, which becomes activated and cleaves pro-IL-1β and pro-interleukin (IL)-18 into mature, secreted forms. Mature IL-1β and IL-18 engage receptors on endothelial cells, immune cells, and other tissues, driving pro-inflammatory cytokine production and immune cell recruitment [32]. A particularly powerful feed-forward loop may be established, where mitochondrial ROS directly oxidizes NLRP3 cysteine residues, enhancing inflammasome assembly and activity [63]. NLRP3 activation produces IL-1β, which stimulates further ROS production in both stressed tissue and recruited immune cells, further activating NLRP3, creating an ROS-NLRP3-IL-1β amplification circuit. This feed-forward loop could sustain systemic inflammation months to years after the initial emotional stressor, explaining the chronic inflammation observed in trauma survivors [24]. However, whether mtDAMP-driven NLRP3 activation is the primary driver versus one contributor among many immune dysregulation mechanisms in PTSD remains unresolved. Chronic systemic sterile inflammation, characterized by elevated circulating IL-1β, IL-6, TNF-α, and type I interferons, may drive immune dysregulation: T cells may shift from regulatory T cell (Treg) and anti-inflammatory Th2 phenotypes toward pro-inflammatory Th1 and Th17 phenotypes; B cell class switching may be biased toward pro-inflammatory IgG subtypes; and tissue-resident macrophages may become chronically activated [25,64]. Over months to years, chronic sterile inflammation is associated with tissue remodeling, fibrosis, vascular dysfunction, and metabolic dysregulation.

### 5.4. Epigenetic Remodeling and Mitochondrial Dysfunction Persistence

A fundamental mechanistic question is whether mitochondrial dysfunction and inflammation persist even after emotional recovery or stressor removal. One proposed mechanism involves epigenetic remodeling, heritable chromatin modifications that do not alter DNA sequence but change gene expression patterns and can persist across cell divisions. The primary mechanism implicates acetyl-CoA, a critical metabolite produced predominantly in mitochondria through pyruvate dehydrogenase and fatty acid oxidation [65]. Acetyl-CoA serves as the universal acetyl donor for histone acetyltransferases, which add acetyl groups to lysine residues on histone proteins. Acetylated histones create open, transcriptionally accessible chromatin (euchromatin) permitting expression of resilience genes encoding antioxidant enzymes, DNA repair proteins, neurotrophic factors, and anti-inflammatory mediators. However, chronic mitochondrial fragmentation and impaired oxidative metabolism may reduce acetyl-CoA synthesis and its export to the nucleus. The hypothesized result is global histone hypoacetylation, condensed chromatin, and transcriptional silencing of mitochondrial biogenesis and resilience genes [34,66].

A second mechanism involves altered Krebs’s cycle intermediate metabolism. α-Ketoglutarate (α-KG) serves as a cofactor for Jumonji-family histone demethylases and Ten-Eleven Translocation (TET) DNA methyltransferases, which actively remove repressive histone marks and DNA methylation [67]. In healthy mitochondria, α-KG is produced efficiently from glutamate. However, when the ETC is severely impaired, Krebs’s cycle flux declines, and α-KG production falls. Simultaneously, succinate and fumarate accumulate because they cannot be efficiently oxidized at Complex II. Accumulated succinate and fumarate directly inhibit α-KG-dependent demethylases, blocking active demethylation [68]. The theorized result is genome-wide hypermethylation of CpG islands in promoters of genes required for mitochondrial biogenesis (PGC-1α, NRF1, NRF2), metabolic flexibility, and cellular resilience [69]. The metabolic signature of persistent mitochondrial dysfunction would, thus, become inscribed in the epigenome through altered acetyl-CoA and Krebs intermediate availability. Even if mitochondrial function partially recovers, the epigenetic “locks” would persist, continuing to suppress resilience genes and promote disease-driving transcription. This mechanism could explain why emotional stress survivors often experience long-term health consequences decades after the traumatic event, and why stressor removal alone does not always reverse the health trajectory.

### 5.5. Multi-System Disease Manifestations of MALT and Evidence Limitations

The mitochondrial dysfunction, mtDAMP signaling, and epigenetic dysregulation proposed to emerge from chronic MALT could manifest across diverse organ systems, each reflecting the particular bioenergetic and redox demands of that tissue.

Cardiac myocytes, which are among the most ATP-demanding cells with mitochondria occupying ~30% of cell volume, appear particularly vulnerable to these bioenergetic shifts. Impaired ATP production from dysfunctional mitochondria could leave cardiomyocytes unable to sustain contractile force or maintain calcium handling during stress, resulting in impaired ventricular function and arrhythmia vulnerability [57,70]. Prolonged emotional stress exposure is associated with new-onset cardiomyopathy in the absence of coronary artery disease, consistent with the hypothesis that mitochondrial dysfunction serves as a link between emotional trauma and cardiac dysfunction.

Beyond the heart, metabolic regulation is equally dependent on mitochondrial integrity, particularly within pancreatic β-cells. These cells depend extraordinarily on mitochondrial ATP production, where ATP-driven closure of K-ATP channels, depolarization, and calcium influx triggers insulin secretion [71]. When mitochondrial ATP production is impaired through MALT, β-cells may fail to sense glucose and cannot mount adequate insulin secretion responses. Chronic mtDAMP-driven IL-1β production may directly impair β-cell survival and function, accelerating β-cell loss. All the above events could result in insulin secretion failure, hyperglycemia, and accelerated progression to type 2 diabetes mellitus (T2DM) [72]. Indeed, severe emotional stress is epidemiologically associated with T2DM onset and poor glycemic control, consistent with the proposed mechanism [73].

In the central nervous system, hippocampal and prefrontal cortex neurons require sustained ATP for synaptic transmission, dendritic spine plasticity, and transcription of neurotrophic genes including brain-derived neurotrophic factor (BDNF). Mitochondrial dysfunction may reduce ATP availability for Na^+^/K^+^-ATPase pumps and synaptic vesicle recycling, impairing neuronal firing and synaptic strength [74]. Chronic mtDAMP-driven neuroinflammation (elevated IL-1β, TNF-α from microglial cells) may impair long-term potentiation and BDNF signaling, blocking synaptic plasticity [24]. Reduced BDNF can counteract neurogenesis in the hippocampal dentate gyrus as well. The cumulative effect could be cognitive impairment, reduced memory formation, and mood dysregulation [75]. Although depression and anxiety are commonly observed in trauma survivors, whether mitochondrial dysfunction would act as a primary driver or one contributor among many remains unresolved.

Concurrently, immune cells exhibit significant metabolic flexibility, shifting between oxidative phosphorylation (used by anti-inflammatory cells) and glycolysis (used by pro-inflammatory cells) depending on activation state [76]. Mitochondrial dysfunction could skew immune cell metabolism toward a pro-inflammatory profile, promoting differentiation toward Th1 and Th17 phenotypes over anti-inflammatory Tregs [77]. Additionally, mtDAMP signaling may directly activate inflammasome-dependent IL-1β and IL-18 production by macrophages and dendritic cells. The result could be immune dysregulation, reduced tolerance, and increased susceptibility to autoimmune and inflammatory diseases [78]. Emotional trauma is epidemiologically associated with autoimmune disease onset, though whether this reflects mitochondrial dysfunction, HPA axis dysregulation, altered vagal tone, or gut dysbiosis remains unclear [79].

Interesting observations regarding these mechanisms emerge from patients with PMDs. These conditions, caused by genetic mutations in nuclear or mitochondrial DNA encoding ETC proteins, display intrinsic mitochondrial dysfunction from birth or early life. Paradoxically, PMD patients do not uniformly exhibit heightened emotional distress, depression, anxiety, or PTSD, despite having severely impaired mitochondrial function [35]. This dissociation suggests that MALT operates through distinct and more complex pathways than genetic PMD, implying that the emotional consequences of MALT may reflect not simply mitochondrial ATP insufficiency but rather the result of multiple events related to stress hormone-induced mitochondrial signaling, mtDAMP release, and epigenetic remodeling.

Critically, the human evidence base for MALT remains incomplete. Most information about this issue comes from animal models and isolated mitochondria. In humans, only a few studies have described associations between emotional stress and circulating mitochondrial biomarkers (GDF-15, cf-mtDNA) or between trauma exposure and altered peripheral blood mononuclear cells (PBMCs) mitochondrial respiration [19,80]. Evidence establishing a causal link over time remains scarce. To date, no longitudinal study has tracked emotionally traumatized cohorts from baseline, measuring mitochondrial biomarkers and functional capacity alongside stress severity and disease progression. Furthermore, significant confounding factors are often overlooked. For instance, are the observed mitochondrial changes truly caused by trauma, or are they secondary to chronic inflammation, poor sleep, malnutrition, or psychotropic medication? We must also consider alternative mechanisms. Could gut dysbiosis or neural signaling mediate the link between stress and disease independently of mitochondrial dysfunction? Finally, does the variability in trauma outcomes reflect underlying differences in mitochondrial vulnerability, such as genetics or metabolic fitness? These questions remain open and call for rigorous prospective research. Table 1 synthesizes the key features of MALT across acute, transition, and chronic phases of stress-related pathophysiology.

## 6. Clinical Applications: Biomarkers and Therapeutic Strategies

Translating the MALT framework into clinical practice requires objective measures of mitochondrial dysfunction and interventions that restore organelle resilience. This section evaluates current approaches for assessing mitochondrial health and modulating stress-induced dysfunction, emphasizing evidence quality and unresolved challenges.

### 6.1. Biomarkers of Mitochondrial Stress and a Proposed Health Index

No single biomarker yet captures the full spectrum of stress-induced mitochondrial dysfunction with sufficient specificity for clinical diagnosis. GDF-15, a cytokine secreted in response to mitochondrial stress, is elevated in individuals with primary mitochondrial disease, aging, and psychiatric conditions including depression and PTSD [80,81]. However, its utility is limited by concurrent elevation in cancer, heart failure, and renal disease. Similarly, cf-mtDNA in plasma reflects mitochondrial membrane rupture and increases acutely during laboratory-induced stress and in trauma-exposed cohorts, but it also rises in sepsis, autoimmune disease, and aging, reducing diagnostic specificity [19,82]. Urinary 8-oxo-dG, a marker of oxidative nucleic acid damage, correlates with systemic oxidative stress but lacks mitochondrial specificity [83].

Direct functional assessment offers greater mechanistic insight. High-resolution respirometry in PBMCs can quantify spare respiratory capacity, which represents the difference between maximal and basal oxygen consumption and reflects the ability to meet increased energy demands [54,84]. Reduced spare capacity is observed in aging, metabolic disease, and psychiatric illness, but the technique requires specialized infrastructure and may not reflect organ-specific mitochondrial function in the brain, heart, or pancreas [85]. Circulating pro-inflammatory cytokines, such as IL-6 and TNF-α, while strongly associated with trauma exposure, represent downstream consequences of mitochondrial damage rather than direct measures of organelle function. A composite Mitochondrial Health Index could integrate multiple domains, including mitochondrial stress signaling (GDF-15), structural integrity (cf-mtDNA), functional capacity (spare respiratory reserve), and inflammatory output (cytokines), to stratify individuals by MALT burden [86]. Such an index might enable objective risk prediction and monitoring of therapeutic response. However, prospective validation in trauma-exposed cohorts, standardization of assays, and establishment of clinically meaningful thresholds are required before clinical implementation, realistically within the next 5 to 10 years of focused research.

### 6.2. Mitochondria-Targeted Therapeutics

Pharmacological strategies to enhance mitochondrial resilience remain largely experimental in the context of emotional stress. Mitochondria-targeted antioxidants, such as MitoQ and SkQs, accumulate within mitochondria and reduce oxidative damage in preclinical stress models, improving both mitochondrial function and behavioral outcomes [87,88]. While MitoQ is the most well-characterized, SkQs (such as SkQ1) are plastoquinone derivatives that have demonstrated a broader therapeutic window and higher potency in some models of aging and oxidative stress, potentially overcoming the pro-oxidant risks associated with high-dose MitoQ. Early-phase human trials in cardiovascular and age-related conditions are ongoing, but efficacy in trauma-exposed populations has not been evaluated. Cardiolipin-stabilizing peptides like SS-31 preserve inner membrane integrity and enhance ETC efficiency; they show promise in heart failure and primary mitochondrial disease trials but lack data in stress-related disorders [62]. NAD^+^ precursors, including nicotinamide riboside, boost cellular NAD^+^ levels and activate sirtuins, promoting mitochondrial biogenesis and antioxidant defenses [89]. These compounds improve metabolic and cognitive parameters in aging and neurodegeneration models and show early benefits in human metabolic studies, though trauma-specific trials are absent [90]. Metformin and ketogenic diets may also enhance mitochondrial flexibility. Metformin acts through mild electron transport chain modulation and AMP-Activated Protein Kinase AMP-Activated Protein Kinase (AMPK) activation, while ketogenic diets provide alternative fuels that reduce oxidative stress [91,92]. However, evidence in emotionally stressed individuals remains preclinical. Critically, no randomized controlled trial has yet specifically enrolled trauma survivors as the primary population and measured objective mitochondrial endpoints before and after intervention. This represents the most significant evidence gap for clinical translation.

### 6.3. Non-Pharmacological Approaches to Enhance Mitochondrial Resilience

**Figure 2:**
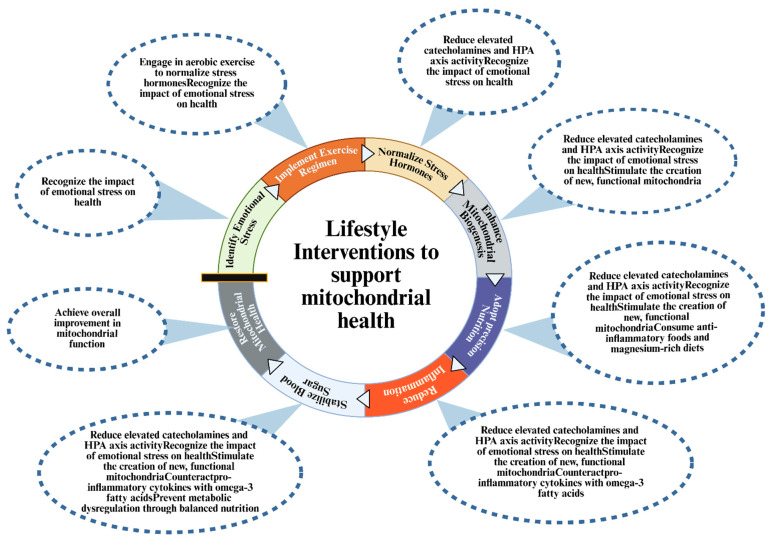
Integrated lifestyle interventions to support mitochondrial health. This infographic illustrates a cyclical strategy for restoring mitochondrial resilience following emotional trauma. The process begins with identifying emotional stress and moves through distinct physiological targets: normalizing stress hormones via aerobic exercise, enhancing biogenesis, reducing inflammation through nutrition, and stabilizing metabolic parameters. Each step builds upon the previous one to achieve overall improvement in mitochondrial function and systemic health. HPA axis: Hypothalamic–Pituitary–Adrenal axis. Created in BioRender. https://BioRender.com/z9qw3d6 (accessed on 10 December 2025).

The culmination of lifestyle and behavioral strategies offers an accessible means to support mitochondrial health. Aerobic exercise robustly enhances mitochondrial biogenesis, spare respiratory capacity, and antioxidant defenses through AMPK and PGC-1α activation [93]. Randomized trials consistently demonstrate that structured exercise reduces symptoms of depression, anxiety, and PTSD, though direct evidence linking these benefits to improved mitochondrial function in trauma survivors is still emerging [94]. Nutritional factors also modulate mitochondrial resilience. Omega-3 fatty acids reduce inflammation and support membrane fluidity, magnesium serves as a cofactor for ATP synthase, and polyphenol-rich foods activate Nrf2-dependent antioxidant pathways [95,96]. Stable blood glucose levels, achieved through balanced macronutrient intake, prevent electron transport chain overload and excessive ROS production. While the mechanistic rationale is strong, trials of comprehensive nutritional interventions in trauma populations are limited. Sleep and circadian regulation are essential for mitochondrial quality control; chronic sleep deprivation impairs biogenesis and increases oxidative stress, while consistent sleep–wake cycles support circadian coordination of metabolic pathways [97]. Psychological interventions, including trauma-focused cognitive behavioral therapy, mindfulness, and social support, reduce HPA axis activation, lower pro-inflammatory signaling, and may indirectly promote mitochondrial recovery by attenuating the neuroendocrine drivers of MALT [98]. Although mitochondrial endpoints are rarely measured in these trials, their effects on stress physiology are consistent with the MALT framework [99]. A structured approach to implementing these lifestyle interventions, targeting specific physiological checkpoints to restore mitochondrial health, is summarized in Figure 2.

### 6.4. Toward Personalized, Mitochondria-Informed Care

Not all individuals exposed to severe trauma develop multi-system illness, highlighting the importance of resilience factors. Genetic variation in antioxidant enzymes, mitochondrial DNA haplogroups, and metabolic regulators may influence individual vulnerability [100]. Baseline metabolic fitness, shaped by physical activity, diet, and early-life programming, likely determines mitochondrial reserve capacity prior to trauma exposure. Cumulative adversity may progressively deplete this reserve, whereas prior mastery of manageable stressors could enhance adaptive responses. Social and psychological resources, including perceived support, meaning, and optimism, predict resilience and are associated with lower inflammation and better stress regulation [101]. Integrating MALT into clinical practice would involve assessing mitochondrial health (via biomarkers or functional assays where feasible), stratifying risk, and personalizing interventions that combine pharmacological support, lifestyle optimization, and psychological healing. If validated, this approach, which positions mitochondrial resilience as a core therapeutic target rather than an adjunct, could represent a new paradigm for trauma-informed medicine [102]. A comprehensive summary of these pharmacological, nutritional, and lifestyle strategies, detailing their specific molecular mechanisms and current clinical evidence, is provided in Table 2.

## 7. Limitations, Challenges, and Considerations

This review offers new perspectives on the pathophysiology of severe emotional stress by proposing mitochondria as central mediators translating psychosocial experiences into systemic disease. By introducing the concept of MALT, we present a transformational framework that honors the fundamental unity of mind and body, positioning mitochondria as the critical convergence point where emotional experiences become biologically embedded. This integrative lens advances our understanding of how subjective psychological distress manifests as objective cellular pathology. Notwithstanding these conceptual innovations, the path from this model to validated science and clinical reality is fraught with substantial limitations and methodological challenges that must be rigorously addressed.

### 7.1. The Challenge of Human-Relevant Models and Causal Inference

A primary limitation is the heavy reliance on pre-clinical models for elucidating core molecular mechanisms. While in vitro studies on cell lines and research in animal models are indispensable for dissecting specific pathways, such as the effect of glucocorticoids on Drp1-mediated fission and their direct translation to the human experience of psychosocial trauma is a formidable leap [103]. Animal models of “stress” often involve acute physical stressors (e.g., restraint or foot-shock) that may not accurately replicate the chronic, cognitively mediated nature of human emotional stress, which is filtered through complex layers of memory, self-identity, and existential distress. The failure of many findings from animal models of inflammation and neuropsychiatric disorders to replicate in humans serves as a stark cautionary tale [104]. Establishing causality in humans is therefore paramount but exceptionally difficult. Specifically, capturing the precise temporal window when adaptive mitochondrial stress responses transition to maladaptive allostatic load remains a logistical challenge, as clinical presentation often occurs months or years after this subcellular shift has taken place. The proposed longitudinal studies are a necessary step, but they cannot entirely eliminate the influence of unmeasured confounding variables, such as diet, physical activity, sleep quality, and environmental exposures, all of which independently affect mitochondrial health [105,106].

### 7.2. The Problem of Biomarker Specificity and Tissue Accessibility

The concept of a “Mitochondrial Health Index” is clinically appealing but faces significant validation challenges. The proposed circulating biomarkers, while promising, lack specificity. Plasma levels of GDF-15, for example, are elevated not only in mitochondrial disease but also in response to cancer, cardiovascular events, and even intense exercise, making it a general marker of systemic stress rather than a specific indicator of MALT [107]. Similarly, levels of cf-mtDNA can be influenced by any process involving cell death or inflammation, such as subclinical infection or injury [108]. Disentangling the signal of psychosocial stress from this background noise will require sophisticated statistical modeling and carefully controlled studies.

Furthermore, a more profound challenge is tissue accessibility. The most clinically relevant organs in the context of stress-related disease are the brain, heart, and endocrine glands. Mitochondrial function in these post-mitotic tissues cannot be assessed directly in living humans. We are therefore reliant on proxy tissues, primarily PBMCs or platelets. The central, unproven assumption is that mitochondrial function in these circulating, short-lived cells accurately reflects the bioenergetic status of critical, long-lived tissues [109]. While some correlation has been demonstrated, the degree to which blood-based mitochondrial measures represent brain or cardiac mitochondrial health remains an area of active and contentious research. This tissue-concordance problem is perhaps the single greatest methodological barrier to validating the MALT hypothesis in humans [110].

### 7.3. Risk of Reductionism and the Complexity of Integration

Finally, this review navigates a complex balance, risking biological reductionism. By focusing intently on the mitochondrion, we risk understating its role as just one component within a vastly complex, multi-scale biological system. The etiology of chronic disease is never attributable to a single molecule or organelle. It is an emergent property of dysregulated networks involving constant cross-talk between the central nervous system, the immune system, the gut microbiome, and the endocrine system, all of which are continuously shaped by an individual’s unique genetic background and life experience [111]. The purpose of this mitochondrial framework is not to replace this complexity with a simplistic, linear model, but to identify a critical, therapeutically tractable node within that larger network. The immense challenge moving forward is one of integration: building computational and conceptual models that can successfully link the dynamics of mitochondrial networks to the functioning of whole physiological systems and, ultimately, to the subjective experience of the individual [112]. Without this successful integration, the insights from mitochondrial biology will remain isolated from the holistic reality of human health and suffering [113].

## 8. Conclusions

The severing of a core social bond is a uniquely human experience with profound biological consequences. The framework presented here argues that this alteration is not confined to the psychological realm but extends to the very core of our cellular biology. We have moved beyond metaphor to propose a specific, mechanistic basis for the “broken heart,” positing that the mitochondrion serves as the critical convergence point where emotional trauma is transduced into physiological collapse.

By introducing the concept of MALT, we provide a testable model that explains the epidemiological link between severe emotional stress and somatic disease. This perspective challenges the foundational dualism of modern medicine, reframing “psychosomatic” or “unexplained” syndromes as predictable, measurable consequences of mitochondrial pathology. The fatigue, pain, and metabolic decay associated with unresolved trauma can now be understood as symptoms of a cellular energy crisis. By identifying the mitochondrion as a shared, druggable target, we open the door to a new class of interventions that can potentially treat both the psychological and physical manifestations of suffering. The central, transformative idea is that healing the mind and healing the cell are not separate endeavors, but two sides of the same coin. To truly understand and treat the consequences of emotional trauma, we must learn to speak the language of our mitochondria.

## Figures and Tables

**Table 1:** Temporal Progression of MALT in Stress-Related Pathophysiology.

Phase	Timeframe	Molecular and Mitochondrial Events	Clinical Features and Biomarkers	Reference
Acute Phase	Minutes to weeks	Catecholamines surge; HPA axis activation; Calcium influx; mPTP opening; Transient ROS burst; Reversible bioenergetic failure	Takotsubo syndrome; Acute stress disorder; Cardiovascular stunning; Troponin elevation; BNP/NT-proBNP; Transient mtDNA release	[41,48,51,54]
Transition Phase	Weeks to months	Glucocorticoid resistance; NF-κB activation; NLRP3 inflammasome assembly; Early fragmentation; Antioxidant depletion; Quality control impairment	Metabolic dysregulation; Persistent inflammation; Early fibrotic changes; Elevated GDF-15; Increased ADMA; Inflammatory cytokines (IL-6, TNF-α)	[62,64,67,70]
Chronic Phase	Months to years	Epigenetic reprogramming; mtDAMP release; Persistent neuroinflammation; Sustained fragmentation; mtDNA damage accumulation; Biogenesis suppression	Multi-systemic diseases: Cardiovascular disorders; Metabolic syndrome; Neurodegeneration; Autoimmune conditions; Chronic cf-mtDNA elevation; 8-oxodG; Reduced mitochondrial respiration in PBMCs	[65,71,74,76]

8-oxodG: 8-oxo-2′-deoxyguanosine; ADMA: Asymmetric Dimethylarginine; BNP: B-type Natriuretic Peptide; cf-mtDNA: Cell-Free Mitochondrial DNA; GDF-15: Growth Differentiation Factor 15; HPA axis: Hypothalamic–Pituitary–Adrenal axis; IL-6: Interleukin 6; mPTP: Mitochondrial Permeability Transition Pore; mtDAMP: Mitochondrial Damage-Associated Molecular Pattern; mtDNA: Mitochondrial DNA; NF-κB: Nuclear Factor Kappa-Light-Chain-Enhancer of Activated B cells; NLRP3: NOD-, LRR- and pyrin domain-containing protein 3; NT-proBNP: N-terminal pro B-type Natriuretic Peptide; PBMCs: Peripheral Blood Mononuclear Cells; ROS: Reactive Oxygen Species; TNF-α: Tumor Necrosis Factor Alpha.

**Table 2:** Mitochondria-Targeted Therapeutic Interventions for Stress-Related Disorders.

Therapeutic Category	Specific Interventions	Mechanism and Targets	Evidence and Considerations	Reference
Lifestyle Interventions	Endurance exercise; High-intensity interval training (HIIT)	AMPK activation → PGC-1α induction; Enhanced mitophagy; Antioxidant enzyme upregulation.	Human trials show improved mitochondrial respiration and stress resilience. Requires consistent adherence.	[93,94]
Nutritional Approaches	Mediterranean diet; Omega-3 fatty acids; Polyphenol-rich foods	Nrf2 pathway activation; Membrane fluidity improvement; Sirtuin activation.	Epidemiological evidence for cardiovascular protection. Compliance challenges in depressed populations.	[95,96,97]
Pharmacological Agents	Mitochondria-targeted antioxidants (MitoQ, SkQ1); SS-31; NAD+ precursors	Direct ROS scavenging at the matrix (MitoQ/SkQ1); Cardiolipin stabilization (SS-31); Sirtuin activation (NAD+).	Preclinical models show cardioprotection. Note: MitoQ has a narrow therapeutic window; SkQ1 shows higher potency in some models. Long-term human safety data needed.	[87,88,89,90]
Psychological Interventions	Mindfulness-based stress reduction; CBT; Trauma-focused therapy	HPA axis normalization; Reduced sympathetic tone; Lowered inflammatory cytokine production.	Randomized trials show reduced inflammation and improved quality of life. Variable response rates.	[98,99]

AMPK: AMP-Activated Protein Kinase; CBT: Cognitive Behavioral Therapy; HIIT: High-Intensity Interval Training; HPA axis: Hypothalamic–Pituitary–Adrenal axis; MitoQ: Mitoquinone; NAD+: Nicotinamide Adenine Dinucleotide; Nrf2: Nuclear Factor Erythroid 2–Related Factor 2; PGC-1α: Peroxisome Proliferator-Activated Receptor Gamma Coactivator 1-alpha; ROS: Reactive Oxygen Species; SkQ1: Skulachev Ion 1; SS-31: Elamipretide.

## Data Availability

Data sharing is not applicable to this article as no new data were created or analyzed in this study. All information discussed is sourced from the publications cited in the reference list.
